# Influencing factors and survival rates in immediate vs. delayed dental implant placement: a six-year retrospective analysis

**DOI:** 10.3389/fdmed.2025.1563641

**Published:** 2025-04-29

**Authors:** Yanfei Cheng, Zhifen Lai, Weiguang Yu

**Affiliations:** ^1^ Department of Oral and Maxillofacial Surgery, Guangdong Provincial Hospital of Traditional Chinese Medicine, Guangzhou, China; ^2^ Department of Radiology, The First Affiliated Hospital, Sun Yat-sen University, Guangzhou, China; ^3^ Department of Emergency Surgery and Orthopedics, The First Affiliated Hospital, Sun Yat-sen University, Guangzhou, China

**Keywords:** survival, factor, dental implant, failure, healed socket, outcome

## Abstract

**Objective:**

This retrospective cohort study aimed to compare survival rates between immediate (≤24 h post-extraction) and delayed (3–4 months post-extraction) dental implants and to identify patient- and site-specific risk factors for implant failure, with emphasis on anatomical site, sex, and osteoporosis.

**Methods:**

We analyzed 1,500 implants (300 immediate, 1,200 delayed) from patients treated at the Guangdong Provincial Hospital of Traditional Chinese Medicine (2005–2023). Kaplan–Meier analysis evaluated cumulative survival rates over 72 months, with Cox regression modeling to assess predictors of failure. Propensity score matching (PSM) addressed baseline covariate imbalances.

**Results:**

Delayed implants exhibited significantly higher survival rates than immediate implants at 72 months (81.1% vs. 53.2%, *p* < 0.0001). Survival divergence intensified after 24 months, with delayed implants retaining 979 patients at risk vs. 202 for immediate implants. Mandibular sites consistently outperformed maxillary sites in both strategies (delayed: 88.5% vs. 72.2%; immediate: 70.5% vs. 40.7%, *p* < 0.0001). Male sex (HR: 1.64, 95% CI: 1.28–1.88; *p* < 0.001) and osteoporosis (HR: 2.50, 95% CI: 1.17–4.52; *p* = 0.024) emerged as independent risk factors, while tobacco use, diabetes, and hypertension showed no significant associations. PSM resolved most baseline imbalances, with post-matching standardized mean differences (SMD) <0.1 for key covariates.

**Conclusions:**

Delayed implantation at 3–4 months post-extraction provides superior intermediate-term survival, particularly in mandibular sites. Male patients and individuals with osteoporosis face elevated failure risks, warranting tailored clinical protocols. While both strategies remain viable, delayed placement is recommended for high-risk populations to optimize long-term outcomes.

## Introduction

Dental implants have become a cornerstone in the rehabilitation of tooth loss, offering functional and aesthetic restoration with high long-term predictability (1–3). However, post-extraction alveolar bone resorption—averaging 5–7 mm horizontally and 1 mm vertically within three months—poses significant challenges for implant placement, often necessitating adjunctive procedures such as ridge preservation with autologous grafts or biomaterials (4–6). Advances in implant surface technology have accelerated osseointegration, enabling diverse placement protocols tailored to post-extraction healing stages (7, 8). Immediate placement (≤24 h) minimizes surgical interventions but carries risks of early failure (9, 10), while delayed protocols (3–4 months) leverage stabilized bone conditions for improved stability (11–13).

**Figure 1:**
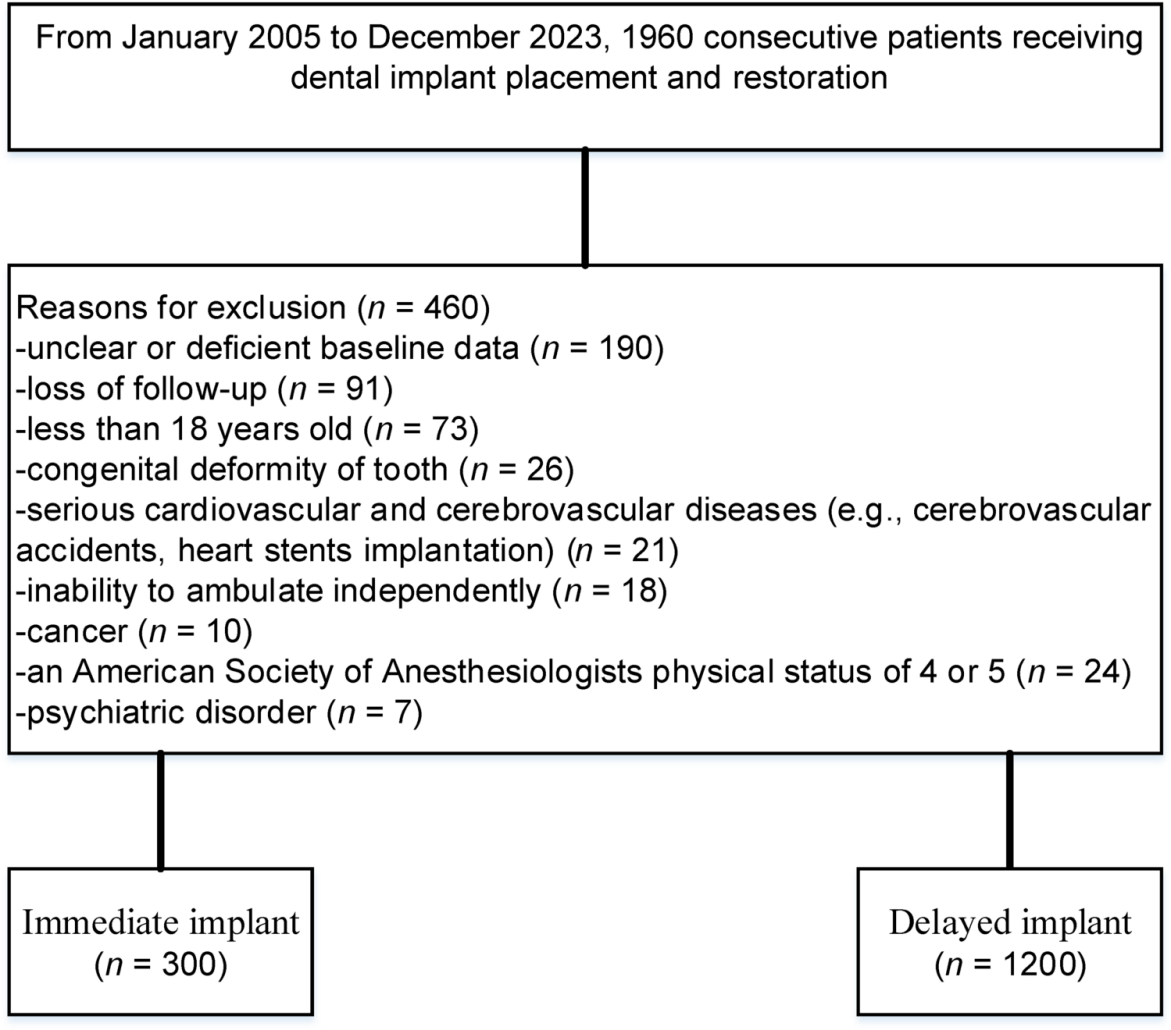
A flow diagram illustrating the methodology for identifying and comparing the survival rates of immediate vs. delayed implants in extraction sockets.

**Figure 2:**
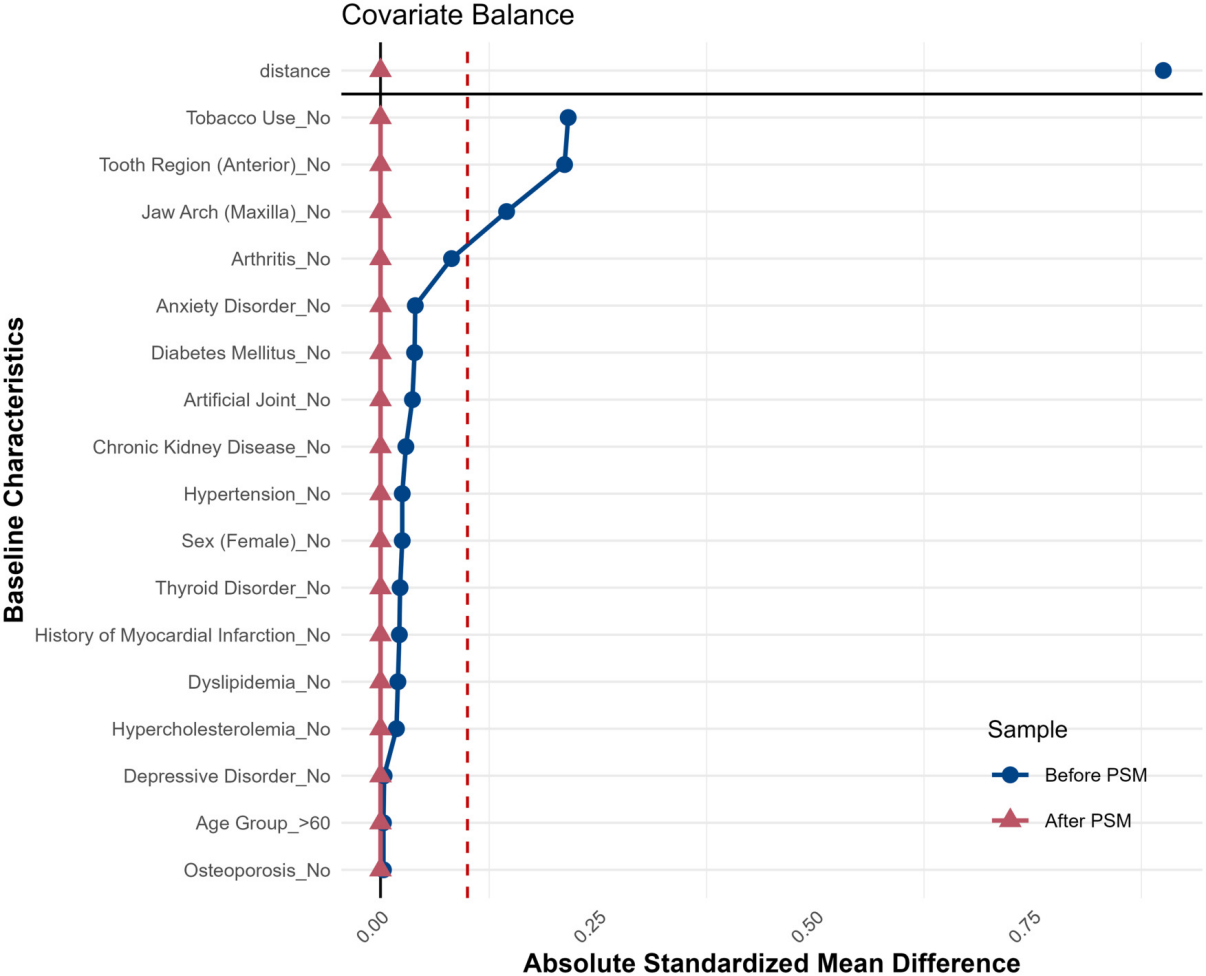
Love plot illustrating SMD for baseline variables before and after PSM. Post-matching SMD values (red) cluster near zero, indicating successful covariate balance.

Beyond biological failures, mechanical complications—such as abutment screw loosening, prosthetic fractures, and peri-implant bone loss under functional loading—are critical contributors to late-stage implant failures (14, 15). These complications often arise from occlusal overload, suboptimal prosthetic design, or inadequate bone-to-implant contact, particularly in compromised anatomical sites (16). While systematic reviews (3, 17) have broadly compared survival rates between immediate and delayed approaches, few studies (18–20) stratify failures by etiology (biological vs. mechanical) or evaluate how placement timing interacts with biomechanical stressors.

Critical gaps persist in the literature: (Ⅰ) limited real-world evidence on delayed implants placed specifically at 3–4 months, (Ⅱ) conflicting data on sex disparities in failure rates (18, 21), and (Ⅲ) unresolved debates regarding osteoporosis as a prognostic factor (1, 2, 4). For instance, while Colak et al. (12) identified male sex as a risk factor for implant loss, others found no significant association. Similarly, osteoporosis remains understudied despite its potential to compromise bone quality (11, 12).

This population-based retrospective analysis addresses these gaps through a modified PICO framework: (Ⅰ) Population: Adults (≥18 years) receiving implants in fresh or healed sockets. (Ⅱ) Intervention: Immediate placement (≤24 h post-extraction). (Ⅲ) Comparison: Delayed placement (3–4 months post-extraction). (Ⅳ) Outcomes: Survival rates, failure risk, and predictors (sex, osteoporosis). By analyzing 1,500 implants placed between 2005 and 2023, with follow-up extending up to 72 months (6 years), this study evaluates intermediate-term outcomes of immediate and delayed dental implant protocols.

## Materials and methods

### Study design and patient eligibility

This retrospective cohort study adhered to the ethical guidelines of the Declaration of Helsinki and received approval from the Medical Ethics Committee of the Guangdong Provincial Hospital of Traditional Chinese Medicine (No. 20/77433). Informed consent was waived due to the anonymized nature of the data and retrospective design. We analyzed electronic dental records of consecutive patients who received dental implants at the Second Affiliated Hospital of Sun Yat-sen University between January 2005 and December 2023, with follow-up extending up to 72 months (6 years). The study included two implant systems: Straumann Bone Level (SLActive® hydrophilic surface) and Nobel Biocare TiUnite® (anodized oxidized surface), with diameters ranging from 3.5 to 5.0 mm and lengths from 8 to 12 mm. These systems were selected based on institutional availability during the 18-year study period (2005–2023). Implant placement and prosthodontic workflows adhered to manufacturer guidelines, with surgeries performed by both faculty surgeons (65%) and supervised residents (35%).

To qualify for inclusion in the study, individuals had to be 18 years of age or older at the time of receiving implant therapy. Additionally, they must have had a comprehensive record of demographic and medical history. The implant therapy must have been administered in university clinics, either by residents or faculty members, and relevant data pertaining to the treatment must have been readily accessible. Patient datasheet, compiled from electronic dental records, included age at implantation, sex, tobacco use, implant characteristics, implant protocol [immediate or delayed (with a delay of 3–4 months)], and systemic conditions.

### Outcomes and assessments

Implant failure criteria followed established guidelines (22, 23), including loss of osseointegration, mobility, pain, fracture, or extensive bone loss (>2 mm). Implant failure was characterized by several criteria, including the loss of osseointegration, the presence of implant mobility, the persistence of pain, the occurrence of implant fracture, or the manifestation of extensive bone loss. Conversely, implant survival was characterized by the stability of the implant and its ability to support restoration at the last follow-up appointment, without the need for explantation. Treatment results were categorized into two distinct groups: survival or failure. Implants were categorized into two groups: immediate (placed ≤24 h post-extraction) and delayed (placed 3–4 months post-extraction). Delayed implants were placed 3–4 months post-extraction, consistent with the Type 3 protocol defined by the European Association for Osseointegration (EAO) for partially healed sockets (24). The follow-ups occurred at 1, 3, 6, and 12 months after the surgical implantation, and after 12 months, follow-ups were conducted every 6 months. The maximum follow-up duration was 72 months (6 years), chosen to evaluate intermediate-term outcomes. Patients with incomplete follow-up (<72 months) were censored at their last recorded visit.

### Statistical analysis

Covariate balance was assessed using propensity score matching (PSM), with an absolute standardized mean difference (SMD) <0.1 considered indicative of adequate balance. Descriptive statistics summarized patient and implant characteristics. Inter-group differences (immediate vs. delayed) were analyzed using *t*-tests for continuous variables and chi-square tests for categorical variables. Survival rates were estimated via Kaplan–Meier analysis, with log-rank tests comparing curves. Survival rates were analyzed at 12-month intervals up to 72 months. Early failures (occurring before prosthetic loading) and late failures (occurring after loading) were stratified. Results at 48 months were emphasized due to the median follow-up period (48 months), while long-term trends were extrapolated to 72 months. Cox proportional hazards regression identified independent predictors of failure, adjusting for age, sex, tobacco use, and systemic conditions. Hazard ratios (HR) and 95% confidence intervals (CI) were reported. Sensitivity analyses excluded patients with incomplete follow-up. All analyses were performed using SPSS v.26.0 (IBM, Armonk, NY) or R software (version, 4.4.2), with *p* < 0.05 considered significant.

## Results

### Cohort characteristics

The study analyzed 1,500 implants (300 immediate, 1,200 delayed) from 1,500 patients (Figure 1). The cohort was predominantly male (90.0%), with 32.3% tobacco users. Immediate implants were more common in the maxillary arch (67.0% vs. 52.5%, *p* < 0.001), while delayed implants predominated in posterior regions (69.2% vs. 48.0%, *p* < 0.001). Tobacco use strongly correlated with delayed protocols (36.6% vs. 15.0%, *p* < 0.001).

PSM reduced SMD, improving covariate balance. Pre-matching SMD ranged from 0.01 to 0.66 (e.g., 0.51 for tobacco use), decreasing post-matching (e.g., 0.255). Most post-matching SMDs were below 0.1, though some variables (e.g., artificial joint, SMD = 0.330; arthritis, SMD = 0.275) retained moderate imbalances. Systemic conditions (diabetes, hypertension, thyroid disorders) showed no significant differences post-matching (*p* ≥ 0.05). Depression prevalence was balanced both pre-matching (16.0% vs. 15.6%, *p* = 0.929) and post-matching (SMD = 0.005). Overall, clinically meaningful imbalances were minimal post-matching, with most covariates achieving adequate balance (Table 1 and Figure 2).

**Table 1:** Baseline characteristics and covariate balance before and after propensity score matching.

Variable	Category	Immediate_Count	Delayed_Count	*p*_value	SMD_Before	SMD_After
Age	18–60 years	226	900	0.964	0.01	0.005
Age	>60 years	74	300	0.964	0.01	0.005
Sex	Female	90	330	0.429	0.06	0.030
Sex	Male	210	870	0.429	0.06	0.030
Tobacco use	Yes	45	439	<0.001	0.51	0.255
Tobacco use	No	255	761	<0.001	0.51	0.255
Arch	Maxilla	201	630	<0.001	0.30	0.150
Arch	Mandible	99	570	<0.001	0.30	0.150
Region	Anterior (incisors, canines)	156	370	<0.001	0.44	0.220
Region	Posterior (premolars, molars)	144	830	<0.001	0.44	0.220
Diabetes	Yes	60	287	0.173	0.09	0.045
Diabetes	No	240	913	0.173	0.09	0.045
Thyroid disorder	Yes	67	241	0.434	0.06	0.030
Thyroid disorder	No	233	959	0.434	0.06	0.030
Kidney disease	Yes	43	207	0.26	0.08	0.040
Kidney disease	No	257	993	0.26	0.08	0.040
High cholesterol	Yes	69	254	0.54	0.04	0.020
High cholesterol	No	231	946	0.54	0.04	0.020
Hypertension	Yes	95	410	0.452	0.05	0.025
Hypertension	No	205	790	0.452	0.05	0.025
History of heart attack	Yes	67	294	0.478	0.05	0.025
History of heart attack	No	233	906	0.478	0.05	0.025
Anxiety	Yes	56	272	0.155	0.10	0.050
Anxiety	No	244	928	0.155	0.10	0.050
Depression	Yes	48	187	0.929	0.01	0.005
Depression	No	252	1,013	0.929	0.01	0.005
Arthritis	Yes	44	274	0.866	0.21	0.105
Arthritis	No	156	926	0.866	0.55	0.275
Artificial joint	Yes	48	236	0.188	0.10	0.050
Artificial joint	No	152	964	0.188	0.66	0.330
Hypercholesterolemia	Yes	84	312	0.479	0.05	0.025
Hypercholesterolemia	No	216	898	0.479	0.06	0.030
Osteoporosis	Yes	96	380	0.967	0.01	0.005
Osteoporosis	No	204	820	0.967	0.01	0.005

SMD, standardized mean difference.

### Survival analysis

**Figure 3:**
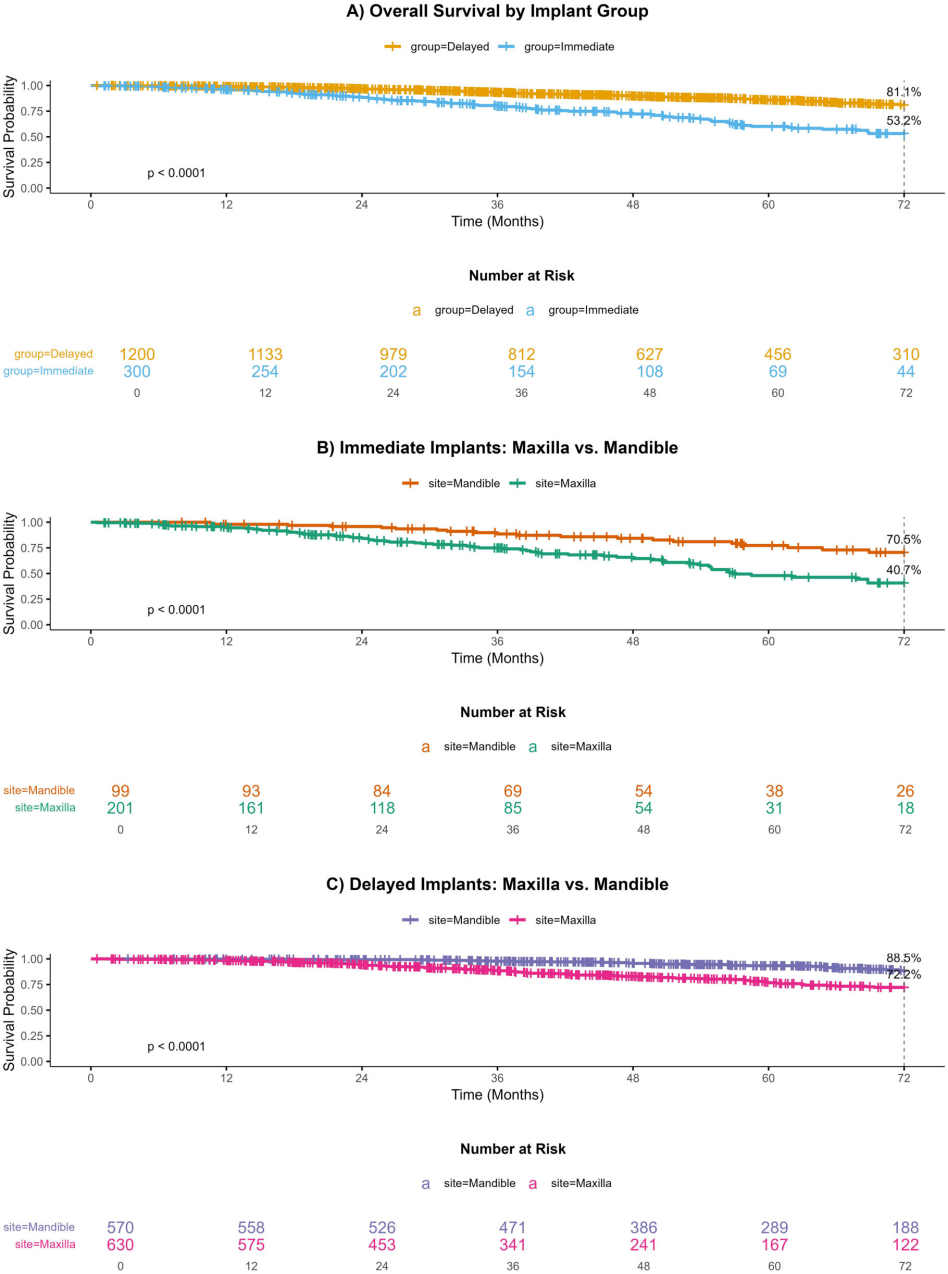
Survival of immediate vs. delayed dental implants in maxillary and mandibular sites: A 72-month Kaplan–Meier Analysis.

The Kaplan–Meier curves demonstrated a significant difference in overall survival between the Delayed and Immediate implant groups (6-year survival rate: 81.1% vs. 53.2%, *p* < 0.0001) (Figure 3A). At 72 months, the Delayed group retained 310 patients at risk (from an initial 1,200), while the Immediate group had only 44 patients remaining (from an initial 300). The survival probability for the Delayed group declined gradually over time, maintaining higher survival rates compared to the Immediate group, which exhibited a steeper decline. These results suggest superior long-term survival outcomes for delayed implants, with a pronounced divergence in survival trajectories after 24 months.

In the Immediate implant cohort (Figure 3B), mandibular implants (*n* = 99 initial) showed a slower decline in survival probability, with 26 patients at risk at 72 months, compared to maxillary implants (*n* = 201 initial), which retained only 18 patients (70.5% vs. 40.7%, *p* < 0.0001). Similarly, for Delayed implants (Figure 3C), mandibular sites (*n* = 570 initial) demonstrated better survival (188 at risk at 72 months) than maxillary sites (*n* = 630 initial; 122 at risk, 88.5% vs. 72.2%, *p* < 0.0001). Despite the larger initial sample size in the maxilla for delayed implants, its survival probability decreased more rapidly, highlighting the mandible's consistent advantage in both implant strategies. These findings underscore the critical role of anatomical location in implant longevity.

### Risk factor analysis

Multivariable Cox regression identified male sex (HR: 1.64, 95% CI: 1.28–1.88; *p* < 0.001) and osteoporosis (HR: 2.50, 95% CI: 1.17–4.52; *p* = 0.024) as independent predictors of failure (Table 2). Males exhibited a 1.6-fold increased risk of implant loss compared to females, while osteoporotic patients faced a 2.5-fold elevated risk. Other variables, including tobacco use, diabetes, and hypertension, showed no significant associations (*p* > 0.05). Cox regression analysis results based on 1,500 samples are shown in Supplementary Table S1.

**Table 2:** Multivariable cox regression model.

Covariate	HR	SE	95% CI lower	95% CI upper	*p-*value
Age	1.065	0.730	0.644	1.174	0.341
Sex (male)	1.641	1.437	1.278	1.879	0.000
Tobacco use	1.213	1.208	0.481	1.700	0.385
Arch: mandible	0.539	2.413	0.344	2.810	0.346
Region: posterior	0.944	1.892	0.568	2.040	0.582
Hypertension	1.485	2.255	0.364	3.684	0.368
History of heart attack	1.058	2.213	0.220	2.664	0.182
Diabetes	2.392	3.214	0.743	4.174	0.462
Thyroid disorder	1.283	1.423	0.619	1.683	0.328
Kidney disease	0.759	1.463	0.409	1.793	0.374
High cholesterol	2.510	3.209	0.576	4.222	0.740
Anxiety	1.623	2.122	0.138	2.604	0.211
Depression	2.188	2.303	0.790	2.707	0.225
Arthritis	1.087	1.381	0.532	1.666	0.362
Artificial joint	1.466	2.038	0.381	2.208	0.175
Hypercholesterolemia	0.651	1.441	0.272	1.756	0.244
Osteoporosis	2.497	3.225	1.167	4.518	0.024

HR, hazard ratio; SE, standard error; CI, confidence interval.

## Discussion

This retrospective cohort study, anchored within a modified PICO framework [Population: adults receiving implants in fresh/healed sockets; Intervention: immediate placement ≤24 h post-extraction; Comparison: delayed placement at 3–4 months (Type 3 protocol, EAO); Outcomes: survival rates and risk factors], analyzed 1,500 implant records to address critical gaps in implant timing protocols and risk stratification. While systematic reviews have broadly compared immediate and delayed implants, our study uniquely focuses on delayed placement at 3–4 months—a timeframe understudied in real-world settings—while concurrently evaluating sex- and osteoporosis-related disparities in failure risk. By integrating these variables into a multivariate model, this work advances personalized decision-making in implantology.

The debate over optimal implant timing persists, with conflicting evidence on survival differences between immediate and delayed protocols (25–27). While randomized trials (28) and prospective studies (29) report comparable short-term outcomes, our data reveal a pronounced divergence in survival at intermediate follow-up (6-year survival: 81.1% delayed vs. 53.2% immediate; *p* < 0.0001). These rates are lower than those reported in recent meta-analyses (30, 31) (e.g., 90%–95% for delayed implants), potentially due to differences in implant systems, operator experience, and patient demographics (32, 33). This aligns with meta-analyses (34) demonstrating elevated failure risks in fresh sockets, particularly in maxillary regions. The progressive survival decline observed in immediate implants beyond 36 months underscores the biomechanical challenges of early placement, such as residual alveolar remodeling and compromised primary stability. In contrast, delayed protocols leverage stabilized bone conditions, potentially mitigating these risks. These findings emphasize the clinical relevance of timing, particularly for high-risk populations.

Notably, delayed implants were disproportionately placed in the mandible (47.5% for delayed implants vs. 33.0% for immediate implants; *p* < 0.001). This anatomic preference may reflect a multifactorial clinical rationale. While the mandible's denser cortical bone historically favors delayed protocols due to enhanced primary stability (35, 36), immediate implants in the maxilla are often prioritized in anterior regions to preserve esthetic outcomes by minimizing soft-tissue collapse (24, 37, 38). Notably, the implants investigated in this study included older-generation systems [e.g., Straumann Bone Level (SLActive® surface) and Nobel Biocare TiUnite®], which may have less advanced surface treatments compared to contemporary designs (39, 40). Systematic reviews (41, 42) corroborate higher maxillary failure rates for immediate implants, attributed to poorer bone quality and greater occlusal forces, whereas delayed protocols in the mandible may capitalize on its biomechanical advantages. However, our multivariate Cox regression adjusted for arch location (HR for mandible: 0.54, *p* = 0.346), confirming that delayed placement retained its survival advantage independent of anatomic site. This suggests that biologic healing processes (e.g., enhanced bone volume and quality) rather than anatomic dominance alone drive the success of delayed protocols.

Our analysis identified male sex (HR: 1.64, *p* < 0.001) and osteoporosis (HR: 2.50, *p* = 0.024) as significant predictors of failure. The heightened risk in males corroborates large-scale retrospective studies (43), which attribute this disparity to biological factors (e.g., bone density variations) and behavioral patterns (e.g., poorer adherence to postoperative care). Additionally, variability in operator experience—65% of procedures were performed by faculty surgeons, while 35% involved supervised residents—may contribute to technical heterogeneity (40, 44). Conversely, conflicting reports (45, 46) suggest that sex differences may be confounded by unmeasured variables like periodontal health, highlighting the need for multifactorial risk models. Similarly, the association between osteoporosis and implant failure—consistent with a comprehensive meta-analysis (3)—reflects systemic bone quality deterioration, which may impede osseointegration. However, the lack of consensus in systematic reviews (47, 48) underscores heterogeneity in osteoporosis management across studies. Our findings advocate for preoperative bone density assessments and targeted interventions (e.g., antiresorptive therapy) in osteoporotic patients to optimize outcomes.

Prior reviews (49, 50) predominantly aggregate heterogeneous protocols, obscuring the nuanced survival patterns associated with specific delayed intervals (e.g., 3–4 months). By delineating this timeframe, our study addresses a critical evidence gap, offering granular insights for guideline refinement. Additionally, the integration of demographic and systemic variables into failure models responds to calls for personalized implantology (51–53), moving beyond a one-size-fits-all approach.

While this study's retrospective design precludes causal inferences and may be influenced by unmeasured confounders (e.g., periodontal health, bone augmentation techniques), its inclusion of real-world, high-risk populations—32.3% tobacco users and 31.7% osteoporotic patients, often underrepresented in controlled trials—provides clinically relevant insights into implant survival under suboptimal conditions. Additionally, the male-dominated cohort (90% male) limits generalizability to female populations, warranting validation in balanced cohorts. These factors likely contributed to lower survival rates compared to idealized cohorts. However, the large sample size (*n* = 1,500), extended 72-month follow-up, and strict adherence to standardized surgical protocols (e.g., consistent implant placement criteria, standardized postoperative care) strengthen internal validity. Furthermore, defining failure as explantation aligns with pragmatic clinical practice, ensuring findings are directly applicable to routine care settings despite inherent limitations of observational data.

## Conclusion

Delayed implantation at 3–4 months post-extraction demonstrates superior intermediate-term survival, particularly in mandibular sites. Male sex and osteoporosis—the latter prevalent in elderly males within our cohort—are critical independent risk factors. While the male-dominated sample limits generalizability to females, our findings underscore the need for sex- and site-specific clinical protocols. Future studies should validate these associations in balanced cohorts and explore biomechanical contributors to posterior maxillary vulnerability.

## Data Availability

The raw data supporting the conclusions of this article will be made available by the authors, without undue reservation.

## References

[1] Karoussis IK Salvi GE Heitz-Mayfield LJA Brägger U Hämmerle CHF Lang NP. Long-term implant prognosis in patients with and without a history of chronic periodontitis: a 10-year prospective cohort study of the ITI® Dental Implant System. Clin Oral Implants Res. (2003) 14(3):329–39. 10.1034/j.1600-0501.000.00934.x 12755783

[2] González JEG Mirza-Rosca JC. Study of the corrosion behavior of titanium and some of its alloys for biomedical and dental implant applications. J Electroanal Chem. (1999) 471(2):109–15. 10.1016/S0022-0728(99)00260-0

[3] Bassir SH El Kholy K Chen CY Lee KH Intini G. Outcome of early dental implant placement versus other dental implant placement protocols: a systematic review and meta-analysis. J Periodontol, (2019) 90(5):493–506. 10.1002/JPER.18-0338 30395355 PMC6500770

[4] Cooper LF Raes F Reside GJ Garriga JS Tarrida LG Wiltfang J Comparison of radiographic and clinical outcomes following immediate provisionalization of single-tooth dental implants placed in healed alveolar ridges and extraction sockets. Int J Oral Maxillofac Implants. (2010) 25(6):1222–32. 21197501

[5] Kim I Ki H Lee W Kim H Park JB. The effect of systemically administered bisphosphonates on bony healing after tooth extraction and osseointegration of dental implants in the rabbit maxilla. Int J Oral Maxillofac Implants. (2013) 28(5):1194–200. 10.11607/jomi.2685 24066308

[6] Becker CM Wilson TG Jensen OT. Minimum criteria for immediate provisionalization of single-tooth dental implants in extraction sites: a 1-year retrospective study of 100 consecutive cases. J Oral Maxillofac Surg. (2011) 69(2):491–7. 10.1016/j.joms.2010.10.024 21238845

[7] Beretta M Maiorana C Manfredini M Signorino F Poli PP Vinci R. Marginal bone resorption around dental implants placed in alveolar socket preserved sites: a 5 years follow-up study. J Maxillofac Oral Surg. (2021) 20(3):381–8. 10.1007/s12663-020-01367-2 34408364 PMC8313622

[8] Debnath A Banerjee S Banerjee TN Paul P. Evaluation of success of dental implants in immediate vs. delayed loading, post radiation in head and neck cancer patients: a systematic review and meta analysis. J Osseointegration. (2024) 16(3):15–26.

[9] El Ebiary SO Atef M Abdelaziz MS Khashaba M. Guided immediate implant with and without using a mixture of autogenous and xeno bone grafts in the dental esthetic zone. A randomized clinical trial. BMC Res Notes. (2023) 16(1):331. 10.1186/s13104-023-06612-8 37957760 PMC10644537

[10] Mahesh L Castro AB Bhasin MT. The survival rate of posterior immediate implants in the maxilla and mandible: an observational retrospective study of 158 dental implants. Cureus J Med Sci. (2023) 15(9):e45579. 10.7759/cureus.45579 PMC10587443 37868567

[11] Chen JY Zhuang MJ Tao BX Wu YQ Ye LJ Wang F. Accuracy of immediate dental implant placement with task-autonomous robotic system and navigation system: an *in vitro* study. Clin Oral Implants Res. (2023) 35(8):973–83. 10.1111/clr.14104 37248610

[12] Çolak S Demïrsoy MS. Retrospective analysis of dental implants immediately placed in extraction sockets with periapical pathology: immediate implant placement in infected areas. BMC Oral Health. (2023) 23(1):304. 10.1186/s12903-023-02986-0 37208620 PMC10197846

[13] Swathi V Kumar NK Anitha R Palachur D Sunder SS Malothu S. Clinical and radiographical evaluation of immediate loading of narrow diameter dental implants. J Pharm BioAllied Sci. (2023) 15:S333–5. 10.4103/jpbs.jpbs_589_22 37654277 PMC10466670

[14] Pjetursson BE Zarauz C Strasding M Sailer I Zwahlen M Zembic A. A systematic review of the influence of the implant-abutment connection on the clinical outcomes of ceramic and metal implant abutments supporting fixed implant reconstructions. Clin Oral Implants Res. (2018) 29(Suppl 18):160–83. 10.1111/clr.13362 30306682

[15] Caballero C Rodriguez F Cortellari GC Scarano A Prados-Frutos JC De Aza PN Mechanical behavior of five different morse taper implants and abutments with different conical internal connections and angles: an *in vitro* experimental study. J Funct Biomater. (2024) 15(7):177. 10.3390/jfb15070177 39057299 PMC11277867

[16] Asmarz HY Magrin GL Prado AM Passoni BB Magalhães Benfatti CA. Evaluation of removal torque and internal surface alterations in frictional morse taper connections after mechanical loading associated or not with oral biofilm. Int J Oral Maxillofac Implants. (2021) 36(3):492–501. 10.11607/jomi.8483 34115063

[17] Pjetursson BE Thoma D Jung R Zwahlen M Zembic A. A systematic review of the survival and complication rates of implant-supported fixed dental prostheses (FDPs) after a mean observation period of at least 5 years. Clin Oral Implants Res. (2012) 23(Suppl 6):22–38. 10.1111/j.1600-0501.2012.02546.x 23062125

[18] Woods B Schenberg M Chandu A. A comparison of immediate and delayed dental implant placement in head and neck surgery patients. J Oral Maxillofac Surg. (2019) 77(6):1156–64. 10.1016/j.joms.2019.02.007 30851250

[19] Nagar P Yadav K Feroz SMA Gangadharappa P Gupta U Loganathan J. Survival and complications of single dental implants in the edentulous mandible following immediate or delayed loading: a randomized controlled clinical trial. J Pharm BioAllied Sci. (2023) 15:S490–4. 10.4103/jpbs.jpbs_554_22 37654368 PMC10466673

[20] Aellos F Grauer JA Harder KG Dworan JS Fabbri G Cuevas PL Dynamic analyses of a soft tissue-implant interface: biological responses to immediate versus delayed dental implants. J Clin Periodontol. (2024) 51(7):806–17. 10.1111/jcpe.13980 38708491

[21] Aragoneses J Jaquez M Rodríguez C Suárez A Aragoneses JM. Early marginal bone loss around immediately loaded one-piece and two-piece dental implants following immediate or delayed loading. A prospective study. J Osseointegration. (2021) 13(1):7–12. 10.23805/JO.2021.13.01.2

[22] Papaspyridakos P Benic GI Hogsett VL White GS Lal K Gallucci GO. Accuracy of implant casts generated with splinted and non-splinted impression techniques for edentulous patients: an optical scanning study. Clin Oral Implants Res. (2012) 23(6):676–81. 10.1111/j.1600-0501.2011.02219.x 21631595

[23] Albrektsson T Zarb G Worthington P Eriksson AR. The long-term efficacy of currently used dental implants: a review and proposed criteria of success. Int J Oral Maxillofac Implants. (1986) 1(1):11–25. 3527955

[24] Hämmerle CH Chen ST Wilson TG Jr. Consensus statements and recommended clinical procedures regarding the placement of implants in extraction sockets. Int J Oral Maxillofac Implants. (2004) 19(Suppl):26–8. 15635943

[25] Anolik RA Nelson JA Rosen EB Disa J Matros E Allen RJ. Immediate dental implant placement in the oncologic setting: a conceptual framework. Plast Reconstr Surg-Global Open. (2021) 9(9):e3671. 10.1097/GOX.0000000000003671 PMC8447991 34548994

[26] Rastogi S Rani K Sharma V Bharti PS Deo K Jain V Osteogenic markers in peri-implant crevicular fluid in immediate and delayed-loaded dental implants: a randomized controlled trial. Clin Implant Dent Relat Res. (2023) 25(3):540–8. 10.1111/cid.13199 36940923

[27] Tumuluri V Leinkram D Froggatt C Dunn M Wykes J Singh J Outcomes of immediate dental implants in vascularised bone flaps for mandibular reconstruction. ANZ J Surg. (2023) 93(6):1682–7. 10.1111/ans.18427 37026415 PMC10953371

[28] Esposito M Zucchelli G Cannizzaro G Checchi L Barausse C Trullenque-Eriksson A Immediate, immediate-delayed (6 weeks) and delayed (4 months) post-extractive single implants: 1-year post-loading data from a randomised controlled trial. Eur J Oral Implantol. (2017) 10(1):11–26. 28327692

[29] Grunder U Polizzi G Goené R Hatano N Henry P Jackson WJ A 3-year prospective multicenter follow-up report on the immediate and delayed-immediate placement of implants. Int J Oral Maxillofac Implants. (1999) 14(2):210–6. 10212537

[30] Chrcanovic BR Albrektsson T Wennerberg A. Reasons for failures of oral implants. J Oral Rehabil. (2014) 41(6):443–76. 10.1111/joor.12157 24612346

[31] Chrcanovic BR Albrektsson T Wennerberg A. Dental implants inserted in fresh extraction sockets versus healed sites: a systematic review and meta-analysis. J Dent. (2015) 43(1):16–41. 10.1016/j.jdent.2014.11.007 25433139

[32] Albrektsson T Wennerberg A. On osseointegration in relation to implant surfaces. Clin Implant Dent Relat Res. (2019) 21(Suppl 1):4–7. 10.1111/cid.12742 30816639

[33] Moraschini V Poubel LA Ferreira VF Barboza Edos S. Evaluation of survival and success rates of dental implants reported in longitudinal studies with a follow-up period of at least 10 years: a systematic review. Int J Oral Maxillofac Surg. (2015) 44(3):377–88. 10.1016/j.ijom.2014.10.023 25467739

[34] Esposito M Grusovin MG Polyzos IP Felice P Worthington HV. Interventions for replacing missing teeth: dental implants in fresh extraction sockets (immediate, immediate-delayed and delayed implants). Cochrane Database Syst Rev. (2010) 9:Cd005968. 10.1002/14651858.CD005968.pub3 PMC12086013 20824846

[35] Kirman CN Tran B Sanger C Railean S Glazier SS David LR. Difficulties of delayed treatment of craniosynostosis in a patient with Crouzon, increased intracranial pressure, and papilledema. J Craniofac Surg. (2011) 22(4):1409–12. 10.1097/SCS.0b013e31821cc50c 21772166

[36] Tevlin R Griffin M Chen K Januszyk M Guardino N Spielman A Denervation during mandibular distraction osteogenesis results in impaired bone formation. Sci Rep. (2023) 13(1):2097. 10.1038/s41598-023-27921-9 36747028 PMC9902545

[37] Crippa R Aiuto R Dioguardi M Nieri M Peñarrocha-Diago M Peñarrocha-Diago M Immediate dental implant placement in post-extraction-infected sites decontaminated with Er,Cr:YSGG laser: a retrospective cohort study. Odontology. (2023) 111(1):255–62. 10.1007/s10266-022-00734-4 36074306 PMC9810677

[38] Morton D Chen ST Martin WC Levine RA Buser D. Consensus statements and recommended clinical procedures regarding optimizing esthetic outcomes in implant dentistry. Int J Oral Maxillofac Implants. (2014) 29(Suppl):186–20. 10.11607/jomi.2013.g3 24660199

[39] Wennerberg A Albrektsson T. Effects of titanium surface topography on bone integration: a systematic review. Clin Oral Implants Res. (2009) 20(Suppl 4):172–84. 10.1111/j.1600-0501.2009.01775.x 19663964

[40] Jemat A Ghazali MJ Razali M Otsuka Y. Surface modifications and their effects on Titanium dental implants. Biomed Res Int. (2015) 2015:1. 10.1155/2015/791725 PMC4575991 26436097

[41] Ghiasi P Ahlgren C Larsson C Chrcanovic BR. Implant and prosthesis failure rates with implant-supported maxillary overdentures: a systematic review. Int J Prosthodont. (2021) 34(4):482–91. 10.11607/ijp.6905 33625390

[42] Gallucci GO Avrampou M Taylor JC Elpers J Thalji G Cooper LF. Maxillary implant-supported fixed prosthesis: a survey of reviews and key variables for treatment planning. Int J Oral Maxillofac Implants. (2016) 31(Suppl):S192–7. 10.11607/jomi.16suppl.g5.3 27228251

[43] Garcia-Sanchez R Dopico J Kalemaj Z Buti J Pardo Zamora G Mardas N. Comparison of clinical outcomes of immediate versus delayed placement of dental implants: a systematic review and meta-analysis. Clin Oral Implants Res. (2022) 33(3):231–77. 10.1111/clr.13892 35044012

[44] Daubert DM Weinstein BF Bordin S Leroux BG Flemmig TF. Prevalence and predictive factors for peri-implant disease and implant failure: a cross-sectional analysis. J Periodontol. (2015) 86(3):337–47. 10.1902/jop.2014.140438 25415249

[45] Patel R Ucer C Wright S Khan RS. Differences in dental implant survival between immediate vs. delayed placement: a systematic review and meta-analysis. Dent J (Basel). (2023) 11(9):218. 10.3390/dj11090218 37754338 PMC10528222

[46] Thanissorn C Guo J Jing Ying Chan D Koyi B Kujan O Khzam N Success rates and complications associated with single immediate implants: a systematic review. Dent J (Basel). (2022) 10(2):31. 10.3390/dj10020031 35200256 PMC8870981

[47] Wu H Shi Q Huang Y Chang P Huo N Jiang Y Failure risk of short dental implants under immediate loading: a meta-analysis. J Prosthodont. (2021) 30(7):569–80. 10.1111/jopr.13376 33932052

[48] Chatzopoulos GS Wolff LF. Dental implant failure and factors associated with treatment outcome: a retrospective study. J Stomatol Oral Maxillofac Surg. (2023) 124(2):101314. 10.1016/j.jormas.2022.10.013 36280552

[49] Young L Grant R Brown T Lamont T. Does a history of periodontal disease affect implant survival? Evid-Based Dent. (2021) 22(1):24–5. 10.1038/s41432-021-0152-8 33772127

[50] Levin L Ofec R Grossmann Y Anner R. Periodontal disease as a risk for dental implant failure over time: a long-term historical cohort study. J Clin Periodontol. (2011) 38(8):732–7. 10.1111/j.1600-051X.2011.01745.x 21635280

[51] Aghaloo T Pi-Anfruns J Moshaverinia A Sim D Grogan T Hadaya D. The effects of systemic diseases and medications on implant osseointegration: a systematic review. Int J Oral Maxillofac Implants. (2019) 34:s35–49. 10.11607/jomi.19suppl.g3 31116832

[52] D'Ambrosio F Amato A Chiacchio A Sisalli L Giordano F. Do systemic diseases and medications influence dental implant osseointegration and dental implant health? An Umbrella review. Dent J (Basel). (2023) 11(6):146. 10.3390/dj11060146 37366669 PMC10296829

[53] Patel R Ucer C Wright S Khan RS. Differences in dental implant survival between immediate vs. delayed placement: a systematic review and meta-analysis. Dent J. (2023) 11(9):218. 10.3390/dj11090218 PMC10528222 37754338

