# Supplementary material for: Influencing factors and survival rates in immediate vs. delayed dental implant placement: a six-year retrospective analysis
Source: Front Dent Med. 2025 Apr 29;6:1563641. doi: 10.3389/fdmed.2025.1563641 (PMC12069371; doi:10.3389/fdmed.2025.1563641)
Supplement: Supplementary file 1 [file Table1.docx]

**Table S1. Cox Regression Analysis Results Based on 1,500 Samples.**

| Covariate | HR | 95% CI | p*-*value |
| --- | --- | --- | --- |
| **Male sex** | 1.64 | 1.28–1.88 | **<0.001** |
| **Osteoporosis** | 2.50 | 1.17–4.52 | **0.024** |
| Age | 1.07 | 0.64–1.17 | 0.341 |
| Tobacco use | 1.21 | 0.48–1.70 | 0.385 |
| Mandibular placement | 0.54 | 0.34–2.81 | 0.346 |
| Posterior region | 0.94 | 0.57–2.04 | 0.582 |

*HR: Hazard ratio; SE: Standard error; CI: confidence interval.*

Sample Size: 1,500 implants (300 immediate, 1,200 delayed).

Covariates: The model included the following variables:

Demographic: Age, sex (male/female).

Behavioral: Tobacco use (yes/no).

Anatomic: Arch (maxilla/mandible), region (anterior/posterior).

Systemic conditions: Hypertension, history of heart attack, diabetes, thyroid disorder, kidney disease, high cholesterol, anxiety, depression, arthritis, artificial joint, hypercholesterolemia, osteoporosis.

Statistical Method: Cox regression was used to assess the independent effects of variables on implant failure risk, reported as hazard ratios (HR) with 95% confidence intervals (CI).
